# Supplementary material for: Development and characterisation of novel oxytocin analogues for PET imaging
Source: Commun Chem. 2025 Nov 5;8:328. doi: 10.1038/s42004-025-01649-1 (PMC12589438; doi:10.1038/s42004-025-01649-1)
Supplement: Supplementary file 2 — Supporting Information [file 42004_2025_1649_MOESM2_ESM.pdf]

Supplementary information: Development and characterization of novel oxytocin analogues for PET imaging

Giancarlo Pascali[A,B,C], Arvind Parmar[A,C], Simone Zanoni[B], Andrew Arthur[A], Anke Hering[D], Ngari Teakle[D], Jack Markham[A,C], Bo Zhang[A,E], Tiffany Mackay[C], Mitch Klenner[A,F], Lawson Spare[A,G], Ivan Greguric[A], Amanda McDonald[A], Aleksandra Bjelosevic[A], Lidia Matesic[A], Gita Rahardjo[A], David Zahra[A], Hasar Hamze[A], Ian B Hickie[C], Richard Banati[A,C], Marie-Claude Gregoire[A,C,H] , Larry Young[I,*], Markus Muttenthaler[D,J] and Adam J. Guastella[C,K].

A: Australian Nuclear Science and Technology Organisation, Sydney, Australia.
B: School of Chemistry, University of New South Wales, Sydney, Australia
C. Brain and Mind Centre, Faculty of Medicine and Health, University of Sydney, Australia
D: Institute for Molecular Bioscience, The University of Queensland, Brisbane, St Lucia, QLD 4072, Australia
E: Rad/Molecular Imaging Program, Stanford University, Stanford (CA), USA
F: Quantum Pharma Australia, Doncaster, VIC, Australia
G: Department of Nuclear Medicine, Liverpool Hospital, Liverpool, Australia
H: Canadian Nuclear Laboratories, Chalk River, Canada
I: Emory University, Atlanta (GA), USA
J: Institute of Biological Chemistry, Faculty of Chemistry, University of Vienna, 1090 Vienna, Austria
K: Children's Hospital Westmead Clinical School, Faculty of Medicine and Health, University of Sydney, Australia

Corresponding authors: G. Pascali gianp@ansto.gov.au; A. Parmar arvind.parmar@sydney.edu.au; M. Muttenthaler m.muttenthaler@uq.edu.au.
* in memoriam († 26 March 2024)

Table of Contents

1. Stability studies, graphs 2
2. Vessel synthesis approach 3
3. Bioconjugation experiments using [^{18}F]ESF 5
4. HPLC profile examples 6
5. Microfluidic setup 10
6. Calibration curve for [^{18}F]dOTK^{8}[SFB] 11
7. PET/CT images 12
8. Evaluation of competition TAC curves 12
9. Explanation of Patlak plot 14
10. Proposed justification for differential blocking results 15
11. LC-MS of OT analogues 16

1. Stability studies, graphs

The graphs reporting the % of intact compound at various time points are shown below.

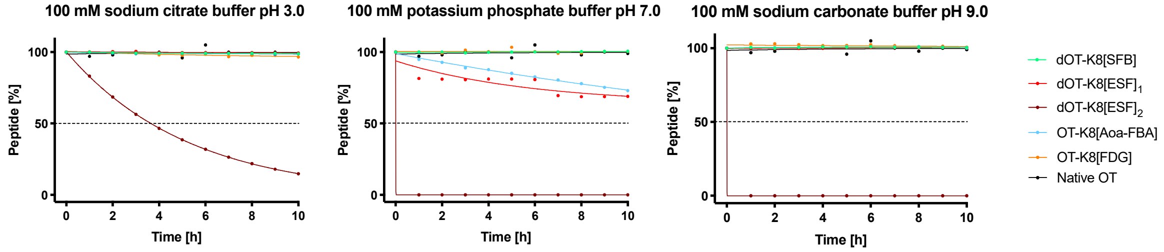

Peptide	$t_{1/2}$		
	**pH 3.0**	**pH 7.0**	**pH 9.0**
OT	>10 h	>10 h	>10 h
dOTK[8][SFB]	>10 h	>10 h	>10 h
dOTK[8][ESF]	>10 h	>10 h	>10 h
dOTK[8][ESF$_2$]	3.6 h	<1 h	<1 h
OTK[8][Aoa-FBA]	>10 h	>10 h	>10 h
OTK[8][Aoa-FDG]	>10 h	>10 h	>10 h

Supp Info 1: pH stability of OT analogues; the results are expressed as mean values of three independent experiments ± SEM.

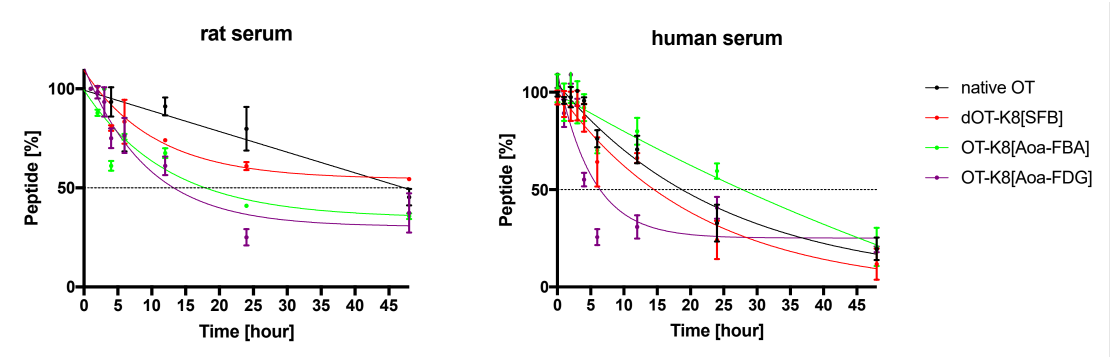

Supp Info 2: Stability study of OT analogues in serums; the results are shown as mean values of three independent experiments ± SEM; corresponding table in the main text.

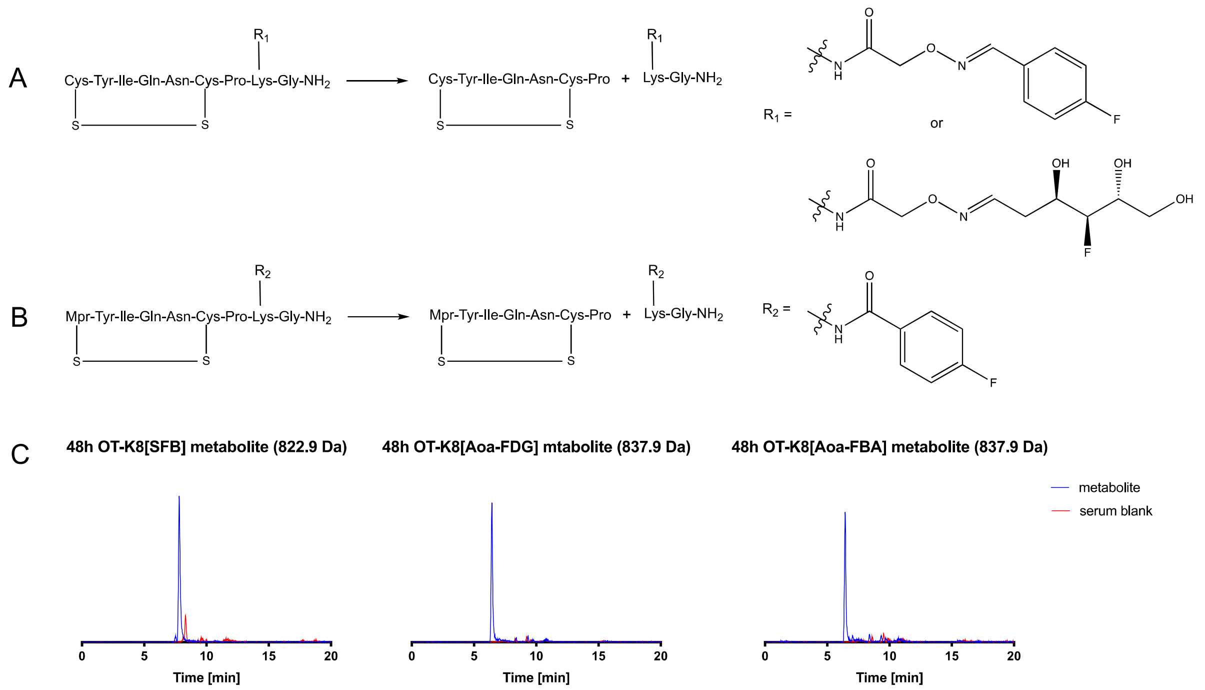

Supp Info 3: Metabolite analysis of dOTK^8[SFB], OTK^8[Aoa-FDG] and OTK^8[Aoa-FBA] in serum. Predicted metabolites for (A) OTK^8[Aoa-FBA] or OTK^8[Aoa-FDG] and (B) dOTK^8[SFB]; (C) MS spectra of measured metabolites for OTK^8[Aoa-FBA], OTK^8[Aoa-FDG] and dOTK^8[SFB]. OT analogues were incubated in rat or human serum for 48 h. Aliquot was taken at 48 h analysed via LC-MS. Each peptide was analysed in three independent experiments, Mpr = dCys. (HPLC column: Agilent Technology, Zorbex C18, 2.1 x 100 mm, 5 µm, 300 Å. HPLC method: 0   50 % B in 50 min, 0.25 mL/min. Solvent A: 0.1 % TFA in H2O. Solvent B: 0.1 % TFA in CH_3CN)

2. Vessel synthesis approach

Synthesis of [^{18}F]SFB (on Synthra)

[^{18}F]SFB was produced using the reaction scheme reported in Supp Info 4. In brief, cyclotron irradiated target [^{18}O]H_2O was passed into a QMA to trap [^{18}F]fluoride; such anion was eluted using a K_2CO_3/K_{222} mixture in ACN/H_2O and the eluate azeotropically dried. The residue was reconstituted with the triflate salt precursor in DMSO, and reaction was performed in a sealed reactor at 90°C for 10 min. Following this, the reaction mixture was passed through a neutral Al cartridge and transferred into a second reactor, where a solution of TPAOH was added to the reactor, and the mixture was heated at 120°C for 5 min. After a quick evaporation of volatiles, the residue was reconstituted with a solution of TSTU and heated at 120°C for 5 min. The reaction mixture was then diluted with 1.5% v/v of AcOH in H_2O and loaded in the loop of a semi-prep HPLC. The product peak was collected ~11.5 min in the employed chromatographic conditions and was further diluted with H_2O before passing into a C_{18} SepPak. The product was eluted with 1.5 mL of ACN and collected in the product vial, where the activity amount was assayed with calibrated detectors; the product was transferred to the microfluidic system (i.e. neighbouring hot cell) for successive steps directly from this vial using N_2 push.

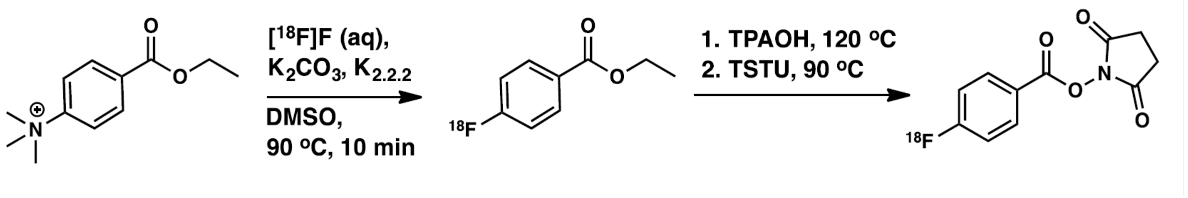

Supp Info 4: Reaction scheme for [^{18}F]SFB production.

The manufacturing process was performed on a Synthra RNplus system loaded with the following conditioned cartridges: QMA Light (V1-V13), Alumina-N Light (V16-V17), and C_{18} Plus (V35-V36). Reagents loaded to the system (Supp Info 5) were:

- A1: 1.0 mL of 2 mg/mL K_2CO_3 and 10 mg/mL K_{222} in ACN:H_2O (80:20)
- A2: 1.0 mL of 1.5% v/v AcOH in H_2O
- A3: 0.5 mL of precursor solution (6.0 mg of solid 4-formyl-N,N,N-trimethyl benzenammonium trifluoromethane sulfonate in 0.5 mL DMSO)
- A4: 0.5 mL of 40 mM tetrapropylammonium hydroxide (TPAOH) in acetonitrile
- A5: 1.0 mL of ACN
- A6: 0.6 mL of 17 mg/mL 2-succinimido-1,1,3,3-tetramethyluronium tetrafluoroborate (TSTU) in ACN
- B4: 1.0 mL of 1.5% v/v AcOH in H_2O
- C1: 3.0 mL of 1.5% v/v AcOH in H_2O
- C2: 1.5 mL of ACN
- C3: 5.0 mL of H_2O
- SPE: 25.0 mL of 1.5% v/v AcOH in H_2O

The final product was eluted through an equilibrated Bondaclone C_{18} 10µm, 300 x 7.88mm column in the following conditions: mobile phase: TFA/H_2O/ACN (0.1:60:40); flow rate: 4 mL/min; detection: scintillation (radioactivity) and UV @ 254 nm.

The activity yield of $[^{18}F]$SFB was 24±3% for a 60-minute synthesis process, determined using Synthra radiodetectors. Radiochemical purity was confirmed to be >95% in initial validation runs by radio-HPLC (Column: Phenomenex, Luna 5µ C_{18} 100Å, 150 x 4.6 mm, 5 µm; Mobile Phase: Acetic acid:water:acetonitrile (0.1:65:35); Flow rate: 1 mL/min). $[^{18}F]$SFB was used directly in the subsequent $[^{18}F]$dOTK^8[SFB] production process without further quality controls.

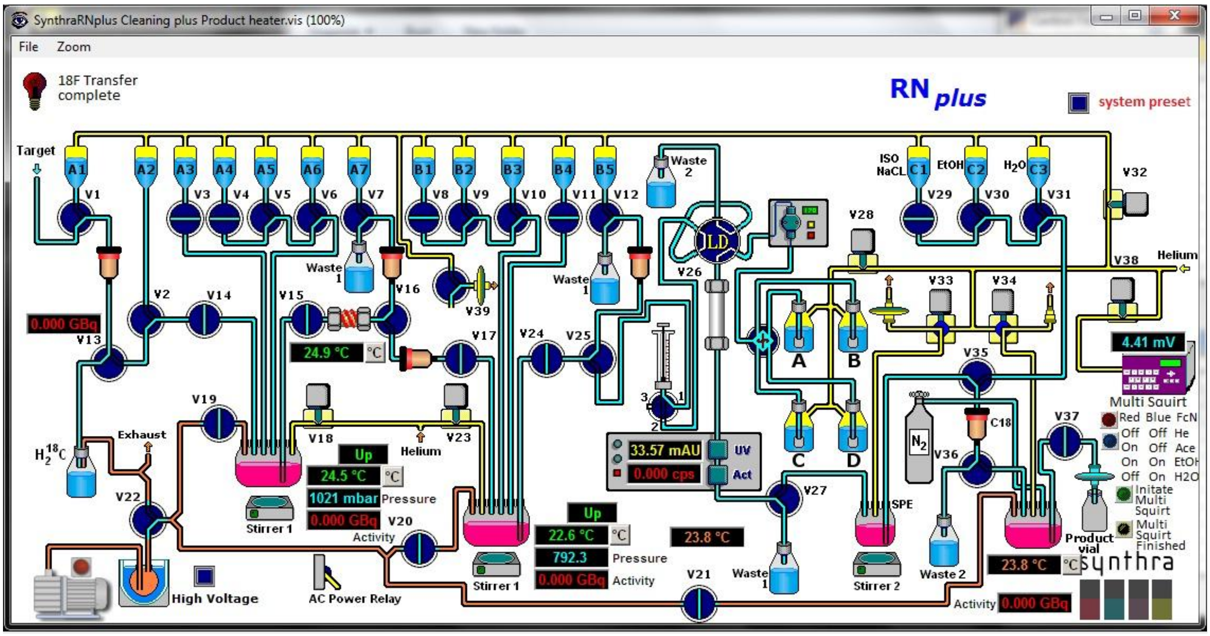

Supp Info 5: Synthra radiosynthesizer schematics.

Radioconjugation reaction, purification and QC analysis (on FlexLab)

The figure below shows the setup of the FlexLab system, loaded and run as described in the manuscript.

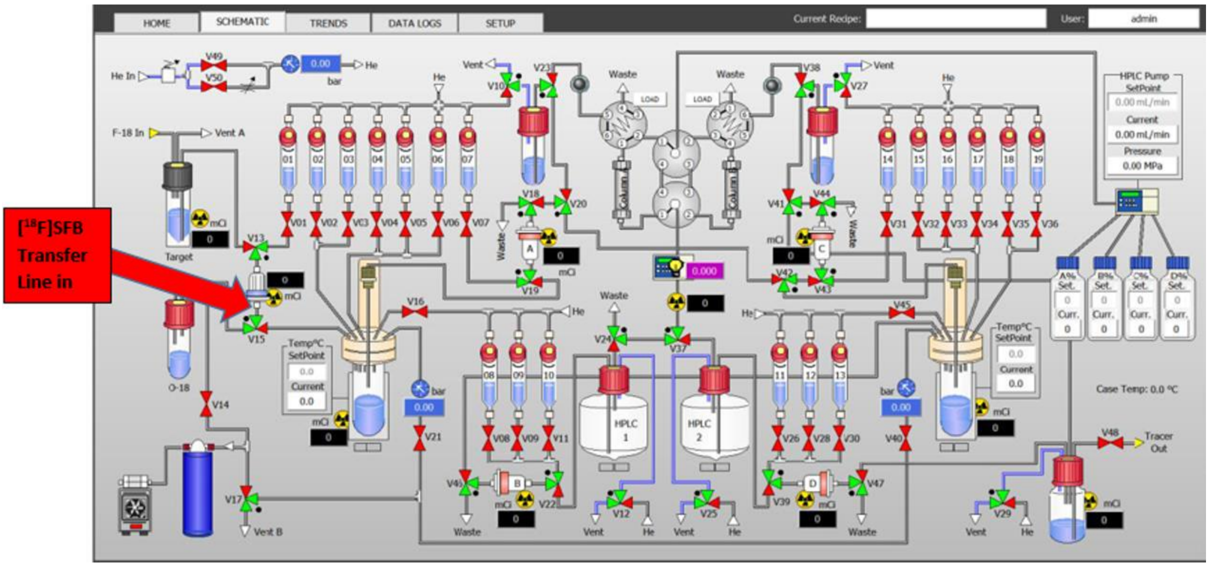

Supp Info 6: FlexLab radiosynthesizer schematics

An alternative QC method by HPLC has been developed as described in the following.

Chemical and radiochemical purity

Instrumentation: Shimadzu UHPLC (SLC-40 controller, LC-40D X3 pump, SIL-40C X3 autosampler, CTO-40C oven, SPD-M40 PDA) and LabLogic FlowRAM NaI well-type radiation detector.

Method: Solvent A – ACN with 0.05% TFA, solvent B – H_2O with 0.05% TFA; wavelength – 220 nm; column – Phenomenex Gemini-NX 150 x 4.6mm; flow – gradient: 10% A (start), 10% A (1 min), 95% A (7 min), 95% A (8 min), 10% A (8.5 min). $[^{18}F]$dOTK^8[SFB] Retention time: 5.4 min.

Molar Activity

Instrumentation: Shimadzu HPLC (CBM-20A controller, LC-20AD pump, SIL-40AC autosampler, SPD-M20 PDA) and LabLogic FlowRAM NaI well-type radiation detector.

Method: Solvent A – 25% ACN, 5% MeOH, 70% H_2O; wavelength – 218 nm; column – Waters Sunfire 150 x 4.6mm; flow – isocratic: 100% A. $[^{18}F]$dOTK^8[SFB] Retention time: 8.7min.

3. Bioconjugation experiments using $[^{18}F]$ESF

$[^{18}F]$ESF was synthesized as carrier added (c.a.) as indicated in the literature. Conjugation reactions were tested directly in an HPLC vial insert, by adding 20 µL of $[^{18}F]$ESF diluted in H_2O to a solution of 1 mg of either dOTK^8 or OT in 80 µL of solvent; 'neat' condition was realized by adding the required volume of $[^{18}F]$ESF and additional volume of 80 µL of H_2O to the dried peptide salts placed in the insert. The obtained mixtures were placed in the autosampler, kept at r.t. and injected in the HPLC analytical system 4 times over ~8 h (Method A, main text).

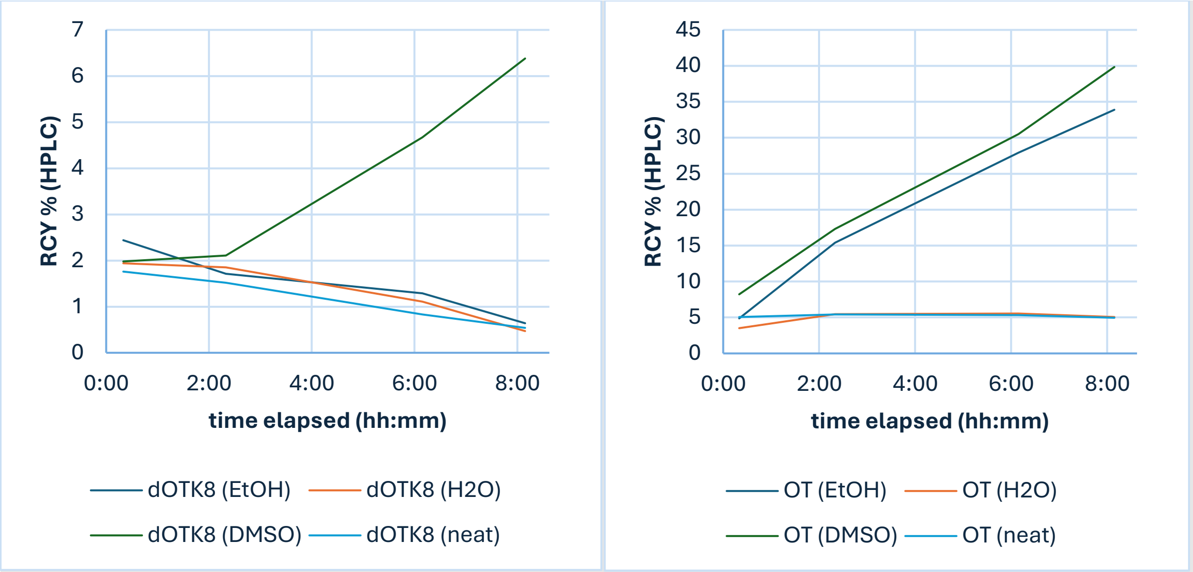

Supp Info 7: RCY of bioconjugation with [^{18}F]ESF, dependence with reaction time and solvents.

From Supp Info 7, it is clear how [^{18}F]ESF reacts faster with OT than with dOTK^8, albeit good yields are obtained only after a few hours at r.t., which is a condition not useful for ^{18}F. For both the peptides, the best solvent is DMSO, while EtOH provides similarly higher yields for OT only. A reduction in yield over time is noticed for dOTK^8, possibly indicating an ongoing degradation of the conjugate in all the solvents except DMSO; these data are aligned with the published instability of ESF adducts. Such degradation is substantially not reported for the OT conjugate.

Given the laboratory setup, for which [^{18}F]ESF was produced using the microfluidic system, no attempts were performed to test the conjugation reaction under microfluidic conditions.

4. HPLC profile examples

This section reports example HPLC profiles of the reaction mixtures for the radioconjugation reactions shown below.

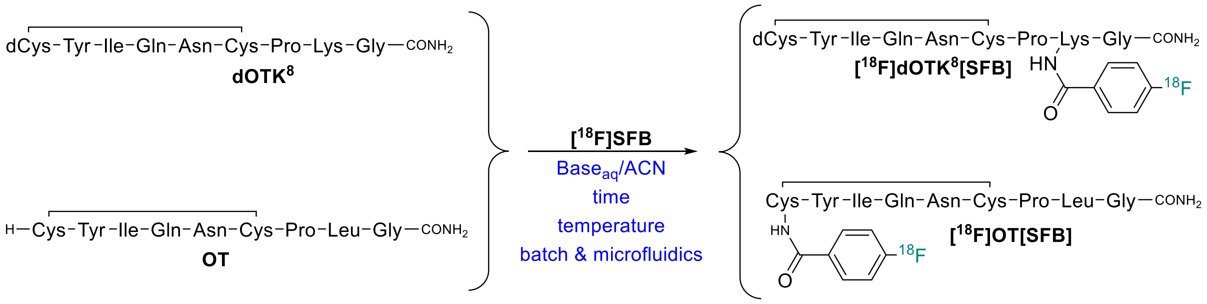

The graphs reported in Supp Info 8 show the product distribution for vessel reactions, and the dependence of RCY with time and temperature. Four radioactive peaks are typically present in the mixture, relating to FBA, SFB, conjugation product and an unknown species; it can be speculated that such unknown species may be due to the cleavage of the disulfide bond, but this hypothesis has not been verified. It is interesting to notice that at 70°C it is possible to consume all the [^{18}F]SFB, as demonstrated by the absence of a late eluting 4th peak in some instances (Supp Info 9).

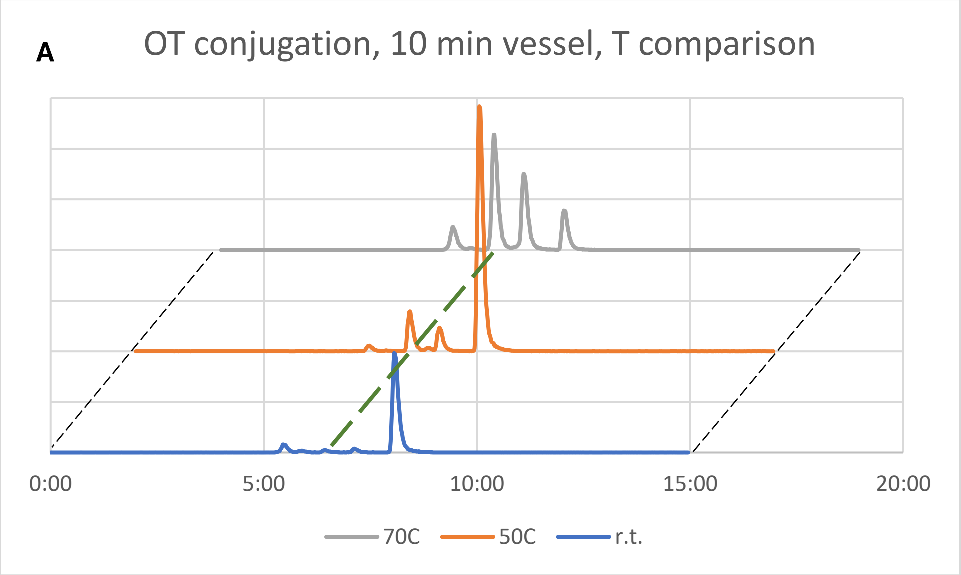

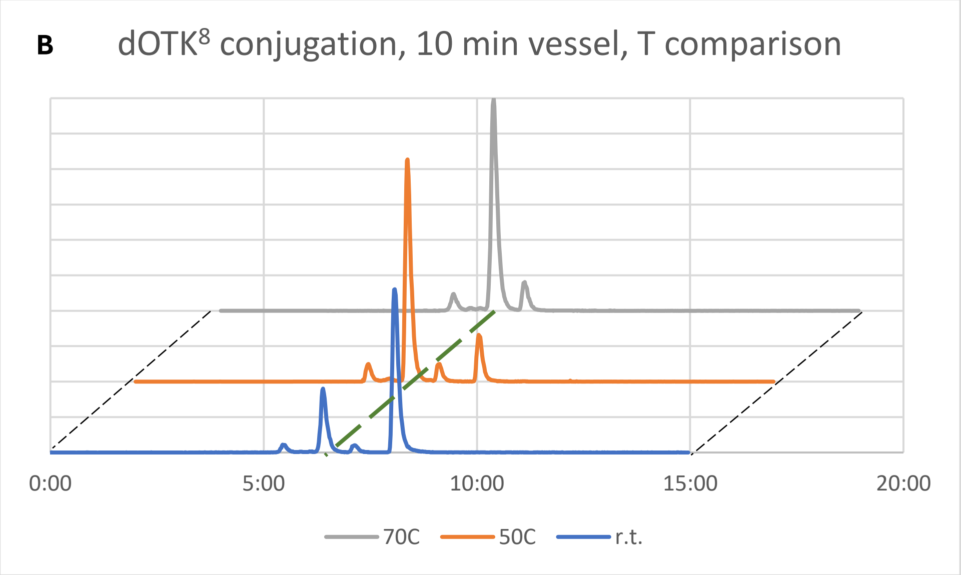

Supp Info 8: HPLC radioactive comparison (Method A, main text) for the [^{18}F]SFB conjugation performed in vessel conditions at three temperatures for 10 min; (A) OT, (B) dOTK^{8}. For both peptides, the 4 peaks are, in order of increasing R_t: [^{18}F]FBA, conjugation product (connected by green line), unknown, [^{18}F]SFB.

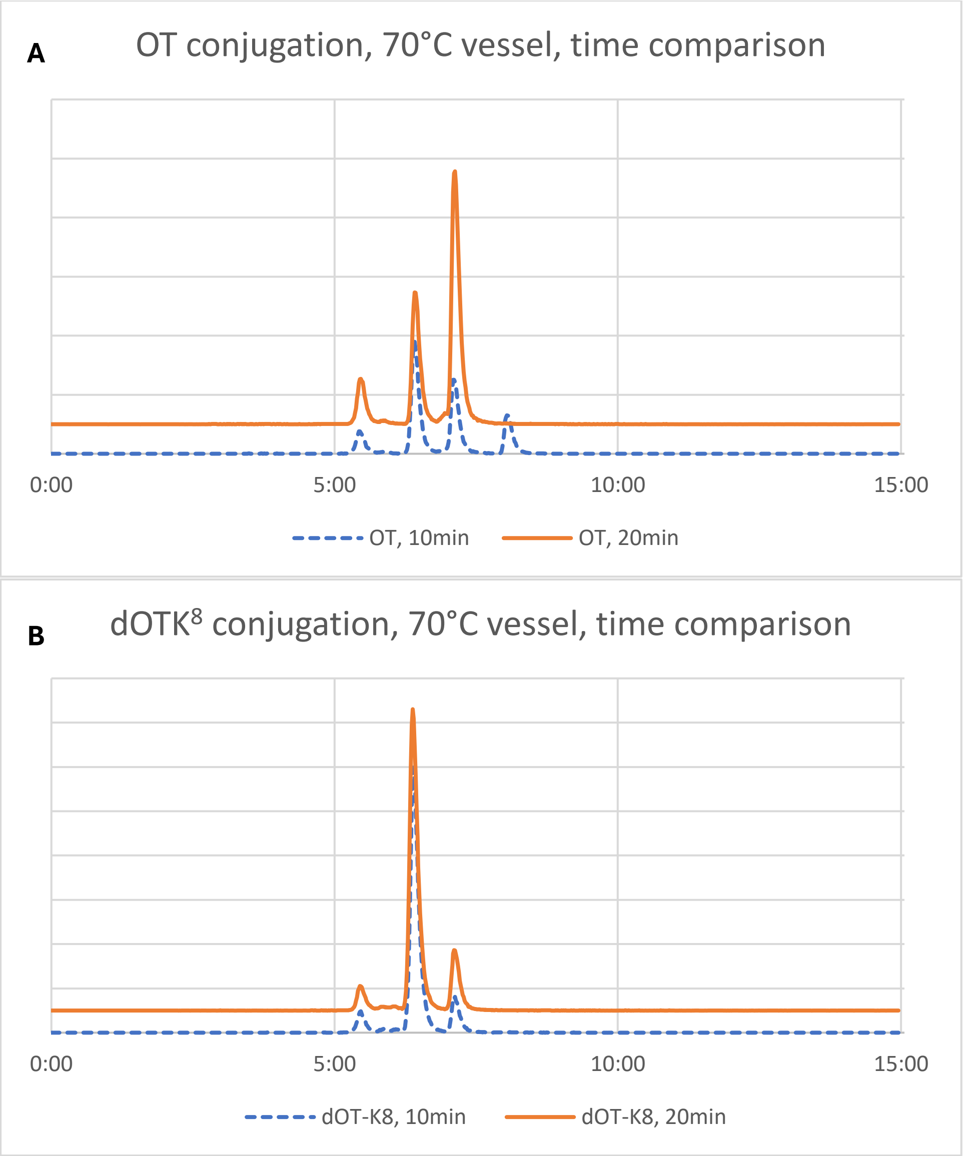

Supp Info 9: HPLC radioactive profile comparison (Method A, main text) for conjugation reactions at 70°C at 10 and 20 min. For both peptides, the 4 peaks are, in order of increasing R_t: [^{18}F]FBA, conjugation product, unknown, [^{18}F]SFB. This last product is only detectable in the reaction with OT for 10min; in all other cases, complete consumption of [^{18}F]SFB is shown by the lack of such 4th peak.

Supp Info 10 shows the radioproducts distribution profile dependency on temperature. It is possible to see how the highest T tested increases the amount of unknown byproduct (i.e. 3rd peak), while the slower flow rate of 5 µL/min increases both the yield of product and byproduct compared to 10 µL/min (Supp Info 11).

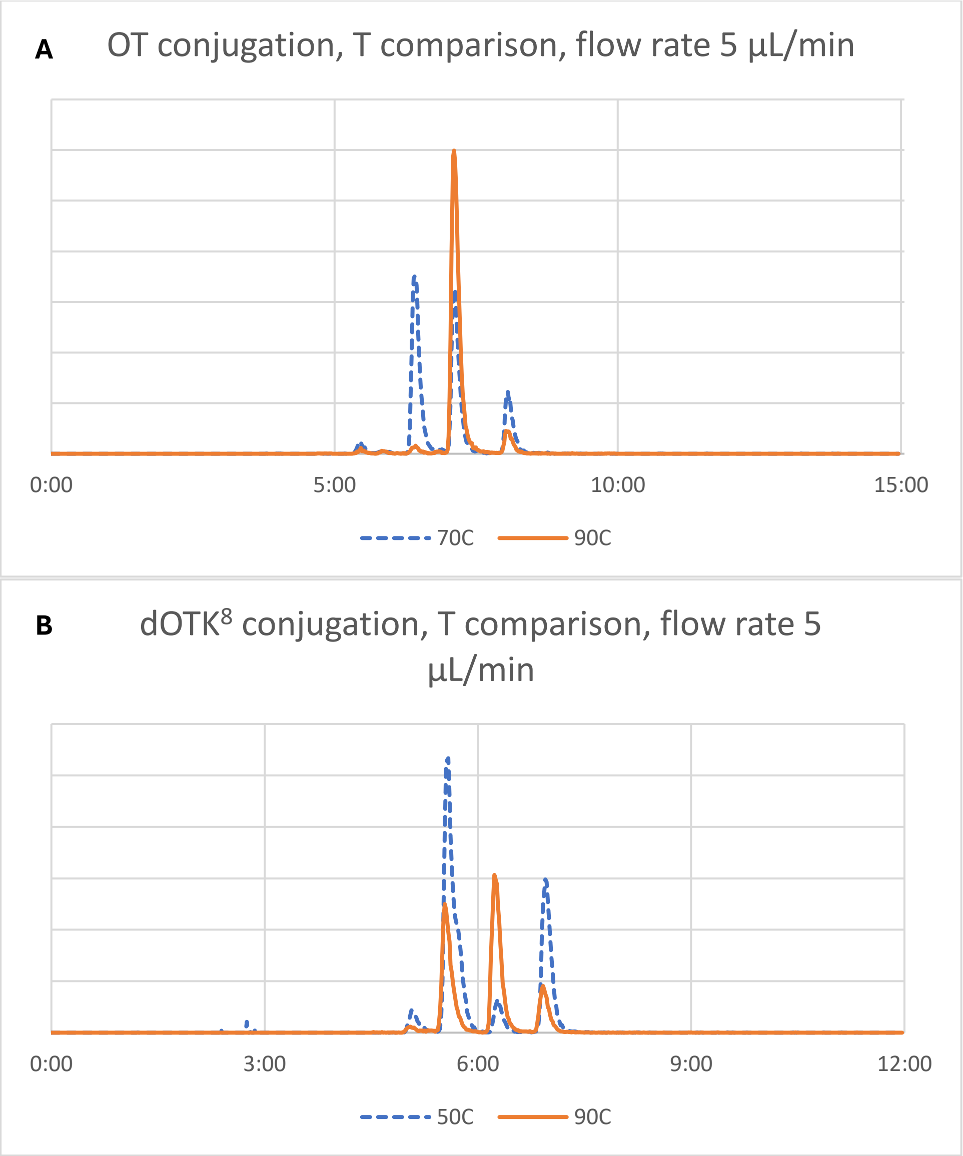

Supp Info 10: HPLC radioactive profile of microfluidic reaction (Method A main text, in panel B the gradient phase was modified to last 12min), showing product distribution dependence on temperature. For both peptides, the 4 peaks are, in order of increasing R_t: [^{18}F]FBA, conjugation product, unknown, [^{18}F]SFB.

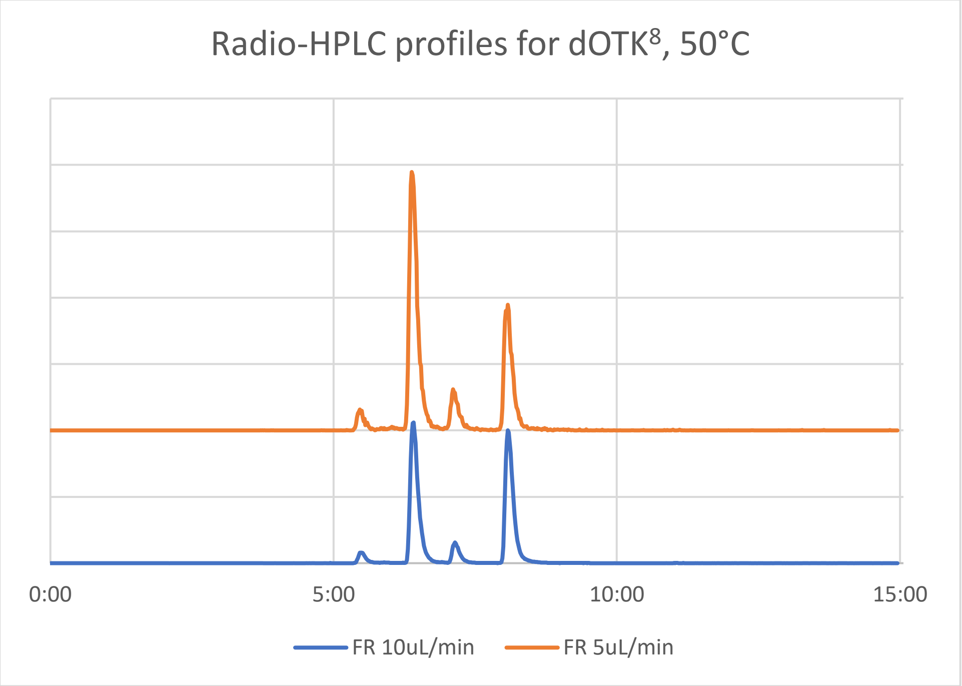

Supp Info 11: HPLC radioactive profile comparison (Method A, main text) using 5 or 10 µL/min flow rate for each channel; reaction on dOTK^{8} at 50°C. The 4 peaks are, in order of increasing R_t: [^{18}F]FBA, conjugation product, unknown, [^{18}F]SFB.

5. Microfluidic setup

Supp Info 12 shows the microfluidic setup used to produce [^{18}F]dOTK^{8}[SFB]. The system has been adapted from previously reported; the key difference was in the formulation steps that, differently from before, it was performed manually. This approach was preferred given the requirement to obtain the smallest final volume possible, in order to allow either intravenous or intranasal administration, although intranasal method was not tested in this study.

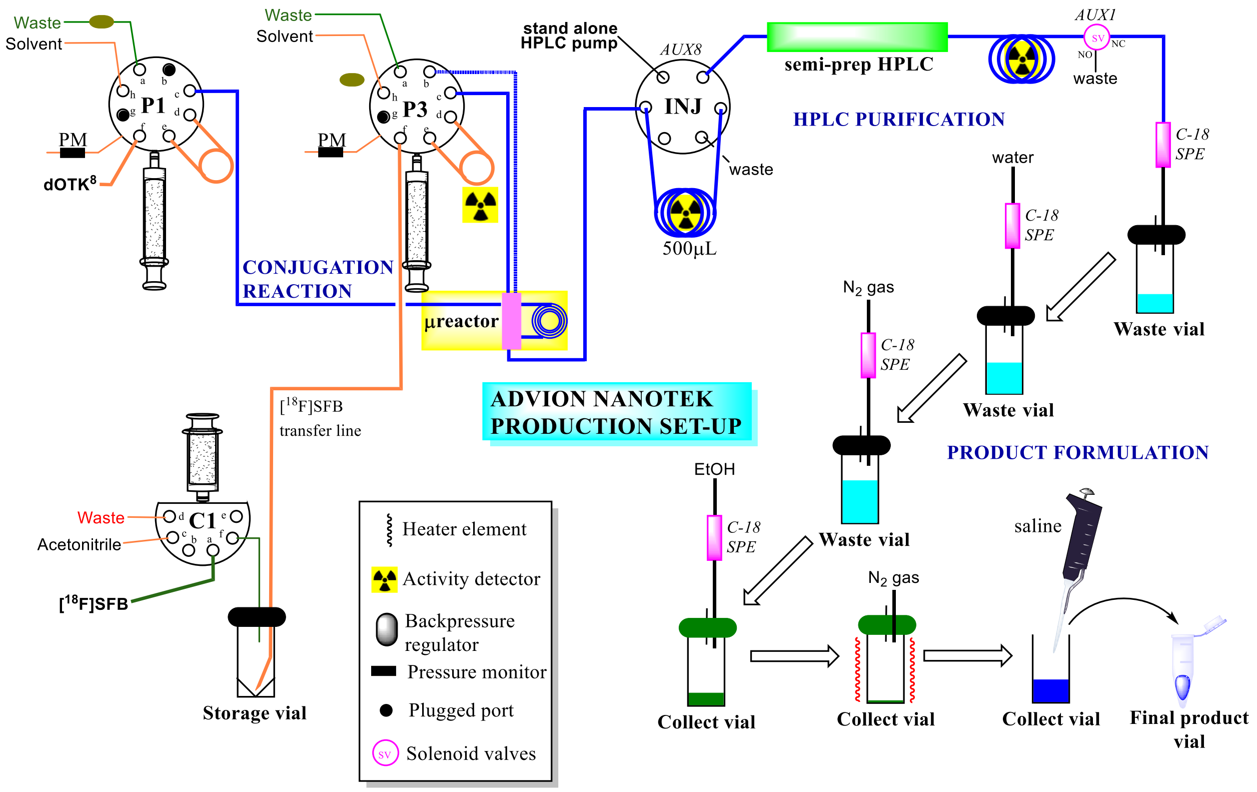

Supp Info 12: Microfluidic system setup for the production of [^{18}F]dOTK^8[SFB].

6. Calibration curve for dOTK^8[SFB]

A fresh calibration curve was recorded before each production campaign. The absolute amount of mass injected to record the curve was in the range 2-1000 ng (2-886 picomoles), while the detected amount for the product was in the range of 10-50 ng.

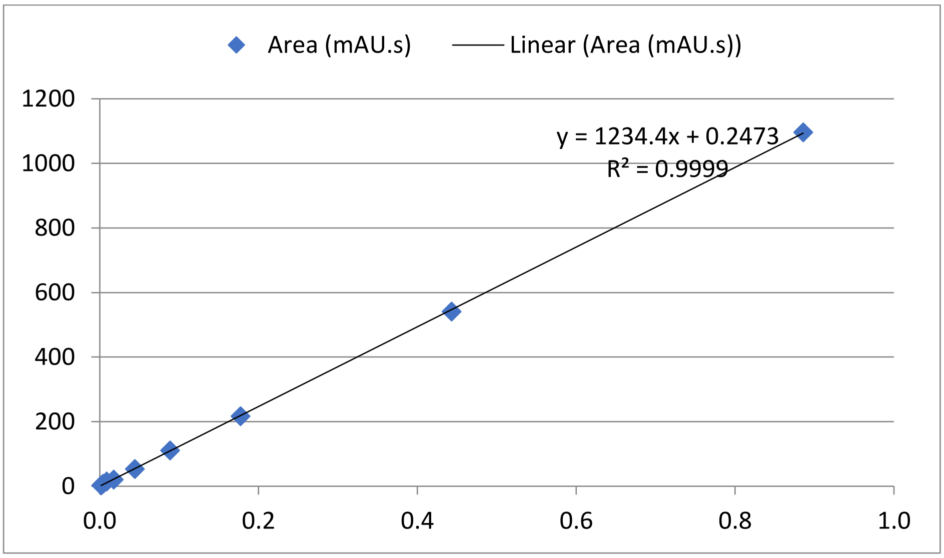

Supp Info 13: Example of calibration curve, fitting equation and correlation value (x-axis: ng injected; y-axis: peak area in UV channel at 215 nm).

7. PET/CT images

An example of PET/CT images planes (coronal, sagittal, transverse; Supp Info 14): mammary pads are evident in the transverse and coronal view (lower set of mammary pads also visible), while pituitary gland is visible at the base of the skull in the sagittal view.

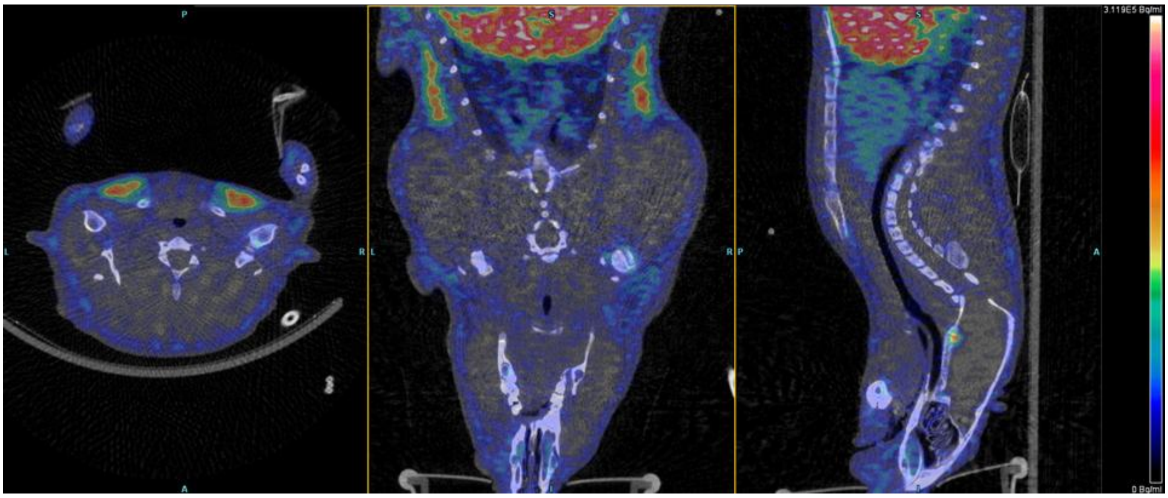

Supp Info 14: Example of PET/CT image on selected planes (from left: transverse, coronal, sagittal view).

8. Evaluation of competition TAC curves

The TACs were analysed to detect a statistically significant displacement of radioactivity (Supp Info 15). To do so, relevant samples of each TAC were selected and categorised into *pre*- and *post*-competitor injection, and a linear regression with categorical covariates was performed. Despite the limitation of having a small number of artifact-free samples after the injection of the competitor, due to the interaction with the animal and its consequent movements, time windows could be consistently identified throughout the different datasets. The outcome of the statistical analysis showed that the difference of the linear regression coefficients between the *pre*- and *post*-estimation was overall not significant ($p < 0.05$) and therefore highlighted the absence of a detectable displacement of radioactivity, confirming the internalization hypothesis.

A linear regression analysis was employed to interpret the two portions of interest of the TAC, i.e. *pre*- and *post*-competitor injection, and the time points of the curves were grouped accordingly (Supp Info 17). To accomplish that, a categorical covariate was used, and thus the regression model included a dummy variable as follow:

$$activity = \beta_0 + \beta_1 t + \beta_2 D + \beta_3 t \times D$$

where β_1 and β_3 are slopes, β_0 and β_2 are intercepts and D is a binary dummy variable indicating the time point grouping. D takes the value 1 if the sample is *post*-competitor injection, 0 otherwise.

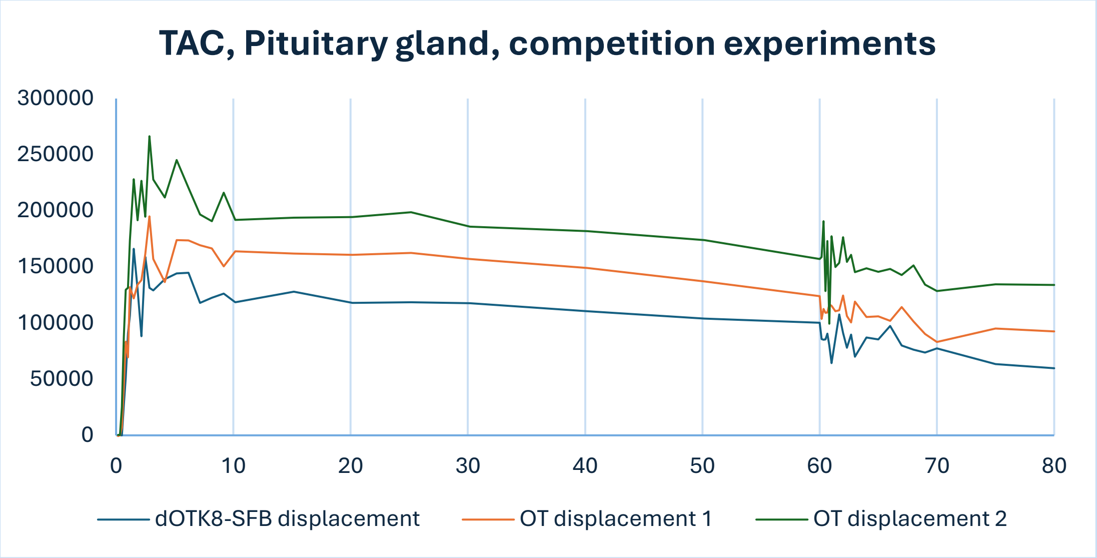

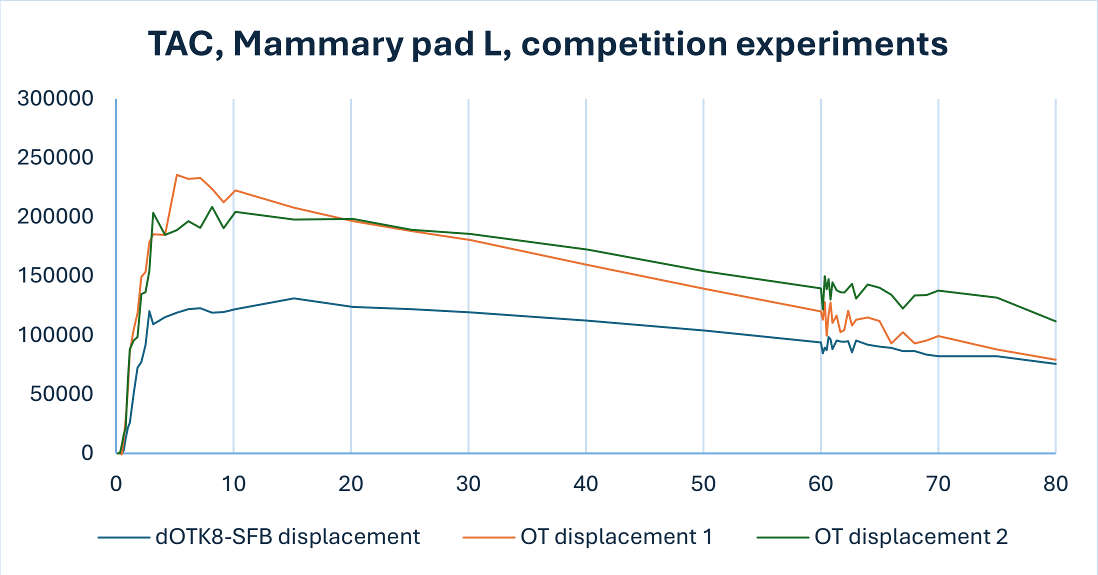

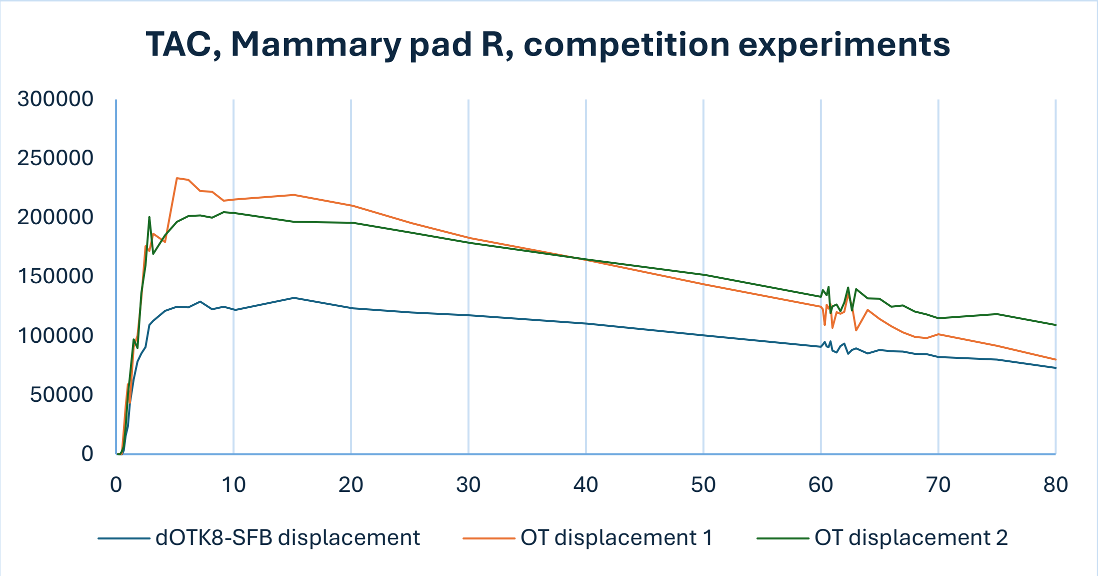

Supp Info 15: Competition experiments testing radiotracer displacement after administering 100 µg of OT or dOTK^{8}[SFB] (data in Bq/mL vs min).

The regression analysis was implemented using the MATLAB function *fitlm* and the returned p-values of the β_2 and β_3 coefficient were interpreted as indices of statistical significance of radioactivity displacement using the 0.05 significance level (**Error! Reference source not found.**).

	β2 p-value - Intercept			β3 p-value - Slope		
	Pituitary Gland	Mammary Pad - Left	Mammary Pad - Right	Pituitary Gland	Mammary Pad - Left	Mammary Pad - Right
R02	0.10995	0.46311	0.86805	0.058828	0.52531	0.88951
R19	0.023765	0.73855	0.10516	0.034054	0.78331	0.18695
R20	0.064476	0.10282	0.15187	0.089443	0.14333	0.15333

Supp info 16: p-values of the β_2 and β_3 regression coefficient in the 3 ROIs (i.e. pituitary gland, left and right mammary pads) of the 3 animals undergoing the competition experiment.

No statistically significant radioactivity displacement was observed across the animals and ROIs, except for the pituitary gland region of the R19 animal.

A possible main limitation of the described approach is represented by the low number of suitable samples in the *post*-competitor injection group due to movement artifacts and opportunities existing to increase the sampling rate, therefore contributing to conduct a more reliable analysis.

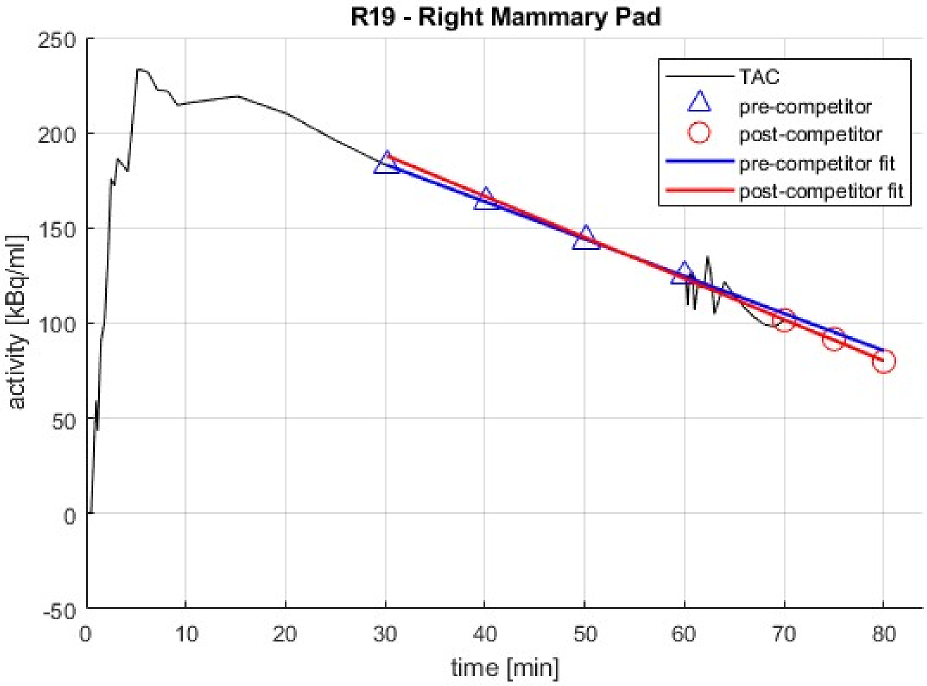

Supp Info 17: Right mammary pad ROI TAC of the R19 animal, pre- and post-competitor time points and linear regressions.

9. Explanation of Patlak plot

The Patlak plot is a graphical analysis method used for the quantification of irreversible kinetics in dynamic PET studies. Originally developed for the quantification of [^{18}F]FDG data (Patlak, C. S., & Blasberg, R. G. (1985). *Journal of Cerebral Blood Flow & Metabolism*, *5*(4), 584-590), it performs a transformation to the PET data such that the estimation of the macro-parameter of interest is reduced to a linear problem. The Patlak plot is given by:

$$\frac{C_{ROI}(t)}{C_P(t)} = K_i \frac{\int_0^t C_P(\tau)d\tau}{C_p(t)} + q$$

where C_{ROI} [kBq/cm^3] is the tracer concentration in the region of interest, C_P [kBq/mL] is the tracer concentration in plasma, q is the ordinate intercept and K_i [mL/cm^3/min] is the irreversible uptake rate constant in tissue, i.e. the macro-parameter of interest.

In receptor studies, such as this one we present, when a region void of specific receptors is available, the Patlak plot expression can be adapted as follow to use such reference region C_{ref} in place of C_P:

$$\frac{C_{ROI}(t)}{C_{ref}(t)} = \frac{K_i}{(V_T' + V_b')} \frac{\int_0^t C_{ref}(\tau)d\tau}{C_{ref}(t)} + q$$

where V_T' is the volume of distribution of the tracer and V_b' is the blood volume of the reference region. For simplicity, the parameter of interest can be referred to as follow:

$$\frac{K_i}{(V_T' + V_b')} = K_i^{ref}$$

The parameter estimation only concerns the Patlak plot portion after a time $t^{\cdot}$, after which the plot becomes linear. However, as the user-dependent choice of $t^{\cdot}$ can affect the K_i^{ref} estimates, we selected different plausible $t^{\cdot}$ and evaluated the SEM of the macro-parameter of interest.

10. Proposed justification for differential blocking results

Following the different timing of internalization, tracer metabolism, resensitization and injection gap, it is possible to hypothesize scenarios for which the blocking study can be successful, whereas an unsuccessful study does not fully demonstrate the lack of the specificity of the tracer.

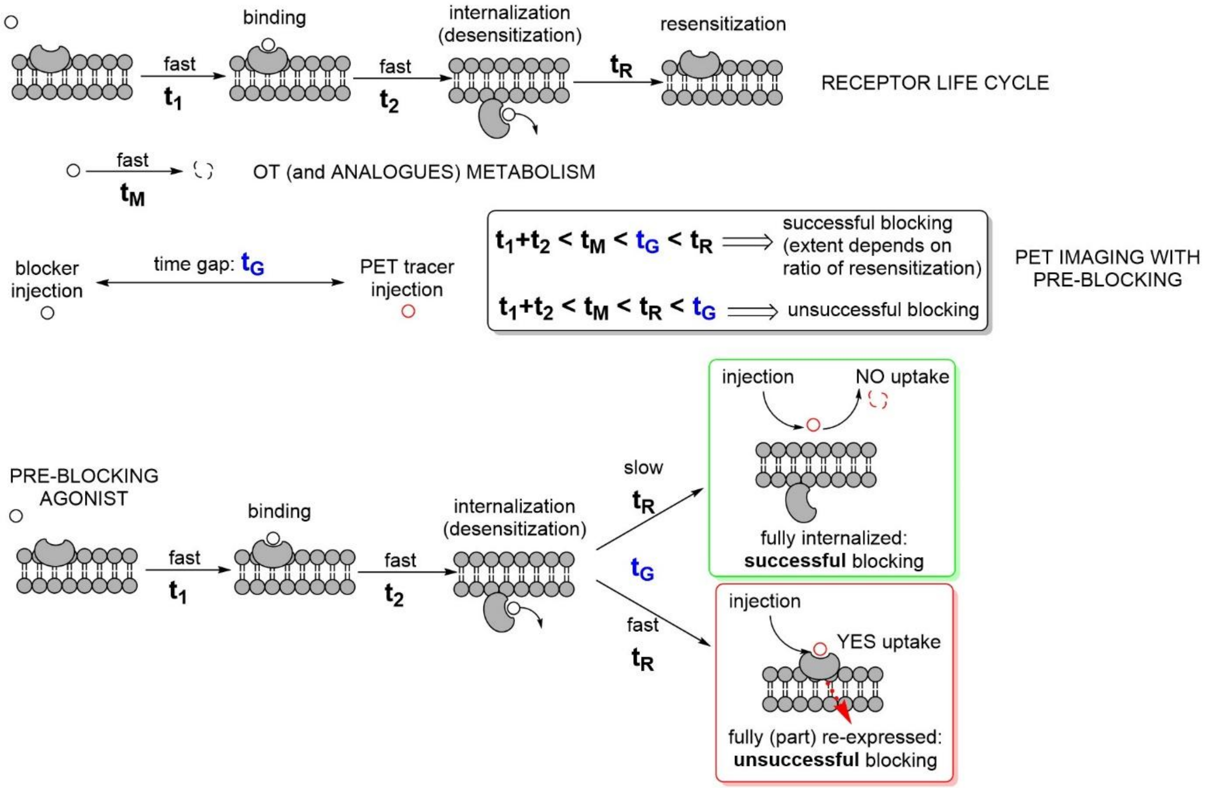

Supp Info 18: Potential blocking scenarios.

11. LC-MS of OT analogues

Peptide – dOT-K8

Sequence: dCYIQNCPKG*

Molecular weight: 1006.7 Da

*C-terminal amide

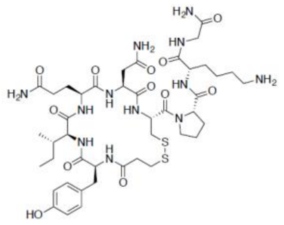

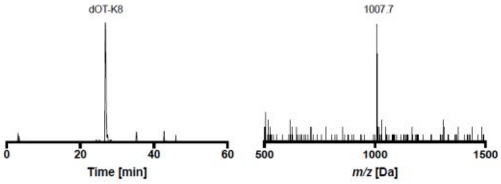

m/z [Da]	ΔM	Comment
1007.7		OT-K8 [M+H]+

Peptide – OT-K8[Aoa]

Sequence: CYIQNCPK[Aoa]G*

Molecular weight: 1096.5 Da

*C-terminal amide

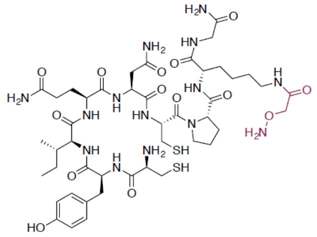

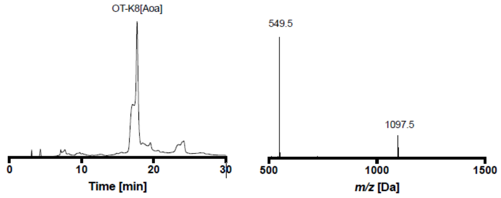

m/z [Da]	ΔM	Comment
1097.5		OT-K8[Aoa] [M+H]+
549.5		OT-K8[Aoa] [M+2H]+2

Peptide - dOT-K8[SFB]

Sequence: dCYIQNCPK[SFB]G*

Molecular weight: 1128.3 Da

*C-terminal amide

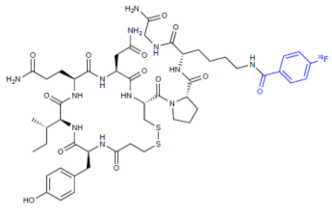

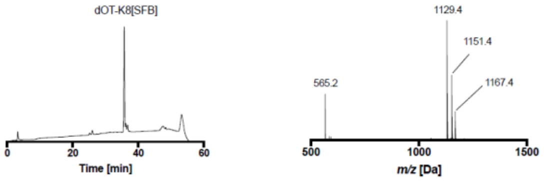

m/z [Da]	ΔM	Comment
1129.4		dOT-K8[SFB] [M+H]+
565.2		dOT-K8[SFB] [M+2H]+2
1151.4	+22	dOT-K8[SFB] + Na+
1167.4	+38	dOT-K8[SFB] + K+

Peptide - dOT-K8[ESF]1

Sequence: dCYIQNCPK[ESF]1G*

Molecular weight: 1116.4 Da dOT-K8[ESF]1

*C-terminal amide

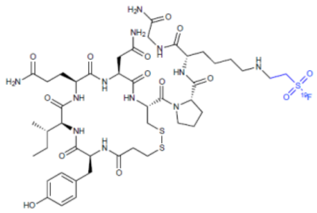

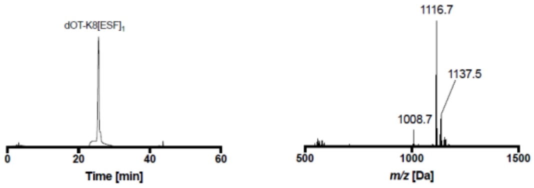

m/z [Da]	ΔM	Comment
1116.7		dOT-K8[ESF]1 [M+H]+
1137.5	+21	dOT-K8[ESF]1 + Na+
1008.7		dOT-K8

Peptide - dOT-K8[ESF]₂

Sequence: dCYIQNCPK[ESF]₂G*

Molecular weight: 1226.6 Da dOT-K8[ESF]₂

*C-terminal amide

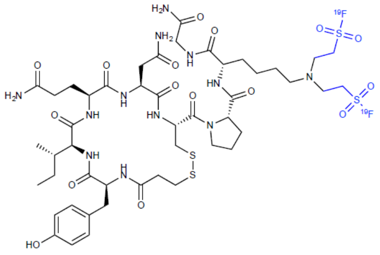

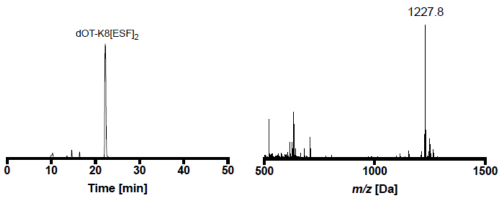

m/z [Da]	ΔM	Comment
1227.8		dOT-K8[ESF]₂ [M+H]⁺

Peptide - OT-K8[Aoa-FBA]

Sequence: CYIQNCPK(Aoa-FBA)G*

Molecular weight: 1200.4 Da

*C-terminal amide

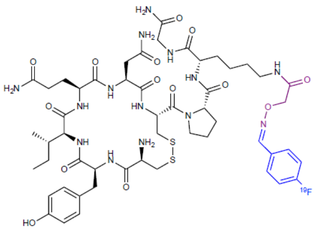

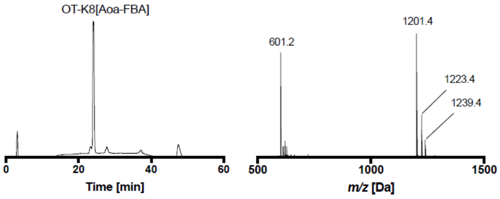

m/z [Da]	ΔM	Comment
1201.4		OT-K8[Aoa-FBA] [M+H]⁺
601.2		OT-K8[Aoa-FBA] [M+2H]⁺²
1223.4	+22	OT-K8[Aoa-FBA] + Na⁺
1239.4	+38	OT-K8[Aoa-FBA] + K⁺

Peptide - OT-K8[Aoa-FDG]

Sequence: CYIQNCPK(Aoa-FDG)G*

Molecular weight: 1259.6 Da

*C-terminal amide

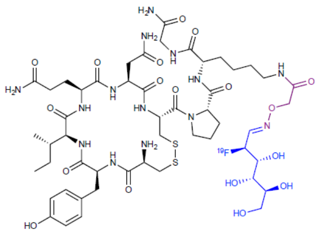

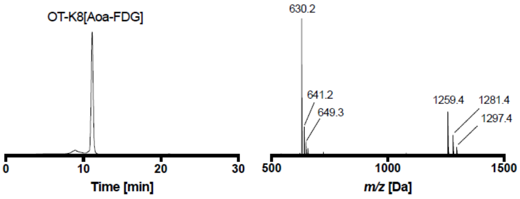

m/z [Da]	ΔM	Comment
1259.4		OT-K8[Aoa-FDG] [M+H]⁺
630.2		OT-K8[Aoa-FDG] [M+2H]⁺²
1281.4	+22	OT-K8[Aoa-FDG] + Na⁺
641.2	+22	OT-K8[Aoa-FDG] [M+2H]⁺² + Na⁺
1297.4	+38	OT-K8[Aoa-FDG] + K⁺
649.3	+38	OT-K8[Aoa-FDG] [M+2H]⁺² + K⁺

Supp Info 19: LC-MS profiles and MS fragmentation spectra of the OT analogues synthesized.

12. cLogP of studied analogues

Comparison of calculated LogP (cLogP) values and experimentally determined HPLC retention times for the OT analogues as performed. While cLogP provides a theoretical estimate of hydrophobicity based on molecular structure, HPLC retention time offers an experimentally derived measure that accounts for the full physicochemical behaviour of the peptides under chromatographic conditions.

Analogue	Retention time	cLogP
OTK^8[Aoa-FDG]	23 min	-4.235
dOTK^8[ESF]	24 min	-1.456
**OT**	**27 min**	**-0.654**
**dOTK^8[SFB]**	**29 min**	**0.134**
dOTK^8[ESF$_2$]	33 min	-0.671
OTK^8[Aoa-FBA]	34 min	-0.659

Supp info 20: Synoptic table of R_t and cLogP for studied analogues, listed in increasing R_t order.
