# Supplementary material for: Development and characterisation of novel oxytocin analogues for PET imaging
Source: Commun Chem. 2025 Nov 5;8:328. doi: 10.1038/s42004-025-01649-1 (PMC12589438; doi:10.1038/s42004-025-01649-1)
Supplement: Supplementary file 3 — Description of Additional Supplementary Files [file 42004_2025_1649_MOESM3_ESM.pdf]

Description of Additional Supplementary Files

File name- Supplementary Video

File description - Example of PET-CT 3D fusion rendering of a baseline experiment
